# NF-kappa B genes have a major role in Inflammatory Breast Cancer

**DOI:** 10.1186/1471-2407-8-41

**Published:** 2008-02-04

**Authors:** Florence Lerebours, Sophie Vacher, Catherine Andrieu, Marc Espie, Michel Marty, Rosette Lidereau, Ivan Bieche

**Affiliations:** 1 Centre Rene Huguenin, FNCLCC, 35 rue Dailly, 92210, St-Cloud, France; 2 INSERM U735, St-Cloud, France; 3 Service d'Oncologie Medicale, Hopital Saint-Louis, 1 Ave C Vellefaux, 75010, Paris, France

## Abstract

**Background:**

IBC (Inflammatory Breast cancer) is a rare form of breast cancer with a particular phenotype. New molecular targets are needed to improve the treatment of this rapidly fatal disease. Given the role of NF-κB-related genes in cell proliferation, invasiveness, angiogenesis and inflammation, we postulated that they might be deregulated in IBC.

**Methods:**

We measured the mRNA expression levels of 60 NF-κB-related genes by using real-time quantitative RT-PCR in a well-defined series of 35 IBCs, by comparison with 22 stage IIB and III non inflammatory breast cancers. Twenty-four distant metastases of breast cancer served as "poor prognosis" breast tumor controls.

**Results:**

Thirty-five (58%) of the 60 NF-κB-related genes were significantly upregulated in IBC compared with non IBC. The upregulated genes were NF-κB genes (*NFKB1*, *RELA*, *IKBKG*, *NFKBIB*, *NFKB2*, *REL*, *CHUK*), apoptosis genes (*MCL1L*, *TNFAIP3/A20*, *GADD45B*, *FASLG*, *MCL1S*, *IER3L*, *TNFRSF10B/TRAILR2*), immune response genes (*CD40*, *CD48*, *TNFSF11/RANKL*, *TNFRSF11A/RANK*, *CCL2/MCP-1*, *CD40LG*, *IL15*, *GBP1*), proliferation genes (*CCND2*, *CCND3*, *CSF1R*, *CSF1*, *SOD2*), tumor-promoting genes (*CXCL12*, *SELE*, *TNC*, *VCAM1*, *ICAM1*, *PLAU/UPA*) or angiogenesis genes (*PTGS2/COX2*, *CXCL1/GRO1*). Only two of these 35 genes (*PTGS2/COX2 *and *CXCL1/GRO1*)were also upregulated in breast cancer metastases. We identified a five-gene molecular signature that matched patient outcomes, consisting of *IL8 *and *VEGF *plus three NF-κB-unrelated genes that we had previously identified as prognostic markers in the same series of IBC.

**Conclusion:**

The NF-κB pathway appears to play a major role in IBC, possibly contributing to the unusual phenotype and aggressiveness of this form of breast cancer. Some upregulated NF-κB-related genes might serve as novel therapeutic targets in IBC.

## Background

The main features distinguishing IBC (Inflammatory Breast Cancer) from other forms of primary breast cancer are a unique phenotype, which includes rapidly progressive breast inflammation, and an extreme tendency to metastasize. The three-year survival rate is about 40%, compared with 85% in non inflammatory breast cancer [1]. The molecular mechanisms underlying these characteristics are largely unknown, but their identification could help with diagnosis, patient stratification and drug development.

We and others have described several molecular alterations in IBC, such as frequent hormone receptor negativity, *TP53 *mutations and *HER2/neu *amplification [2-5]. *In vitro *and *in vivo *studies have implicated *RhoC*, *MUC1*, *E-cadherin *and *LIBC/WISP3 *in the pathogenesis of IBC. The expression of some of these genes is altered in human IBC tumors [6]. However, none of these genetic alterations is specific to the particular phenotype of IBC.

The advent of novel analytical methods such as DNA microarrays has helped to identify molecular signatures for various malignancies. In non inflammatory breast cancer, DNA microarray-based studies have distinguished tumor subclasses with distinct prognoses [7,8]. Few DNA microarray-based studies have been performed in IBC [9-11]. One such study identified a set of 109 genes whose expression discriminated 37 IBCs from 44 non IBCs [9]. These 109 genes, some of which were NF-κB-related, were mainly associated with signal transduction, cell motility, invasion, angiogenesis and local inflammatory processes. Another genome-wide expression profiling study comparing 16 IBCs with 18 non stage-matched non IBCs identified a large number of overexpressed NF-κB-related genes [10]. Using real-time RT-PCR, immunohistochemistry and NF-κB-DNA-binding assays, the same authors recently confirmed the contribution of some of these NF-κB-related genes in IBC [12]. In a previous study of IBC, in which we analyzed the expression of 538 cancer genes by using real-time RT-PCR, we also observed abnormal expression of several NF-κB-associated genes [13].

NF-κB-regulated genes are involved in invasiveness, proliferation, angiogenesis, lymphangiogenesis and inflammation, and are therefore good candidates for explaining the particular characteristics of IBC [14,15]. Increasing evidence suggests that NF-κB-associated pathways are dysregulated in numerous malignancies, including breast cancer [16-20].

To confirm the role of NF-κB target genes in IBC tumorigenesis, we focused on 60 key genes involved in the NF-κB pathway [14,15,21]. We chose real-time quantitative RT-PCR to measure the expression levels of these 60 genes in a well-characterized series of 35 human IBC samples relative to a series of 22 non IBC tumors and 24 distant metastases of breast cancer ("poor prognosis" controls).

## Methods

### Patients and samples

The IBC samples were surgical biopsy specimens obtained from 35 women treated at Saint-Louis Hospital, Paris, France, between 1988 and 1995. IBC was diagnosed on the basis of rapidly progressive signs such as localized or generalized induration, redness and edema of the breast (stage T4d in the 1977 UICC classification). The 35 IBCs were also classified using a staging system named 'Poussee Evolutive' (PEV) developed by Gustave-Roussy investigators in an attempt to refine prognostication in IBC. This staging system takes into consideration aggressiveness of the tumor and signs of inflammation [22]. Using this system, both PEV2 and PEV3 are consistent with IBC. In 13 patients the entire affected breast was inflammatory (stage PEV3), while in 22 patients the inflammation was localized (stage PEV2).

All biopsies were performed before treatment, and infiltrating carcinoma was documented histologically in every case. All the patients underwent first-line high-dose anthracycline-based chemotherapy followed by local treatment. At the time of this analysis, 26 patients had relapsed and 9 remained disease-free. Each patient gave written informed consent. The Local Ethical Committee approved this study.

As "non IBC" controls, we used specimens of 22 non inflammatory locally advanced breast cancers (LABCs), of which 6 were stage IIb and 16 were non inflammatory stage III. These 22 non IBC controls were all high-grade invasive ductal carcinomas (Scarff-Bloom-Richardson histopathological grade III). The mRNA levels of the 60 genes in IBCs were expressed relative to those in non IBCs.

As "poor prognosis breast tumor controls" we used biopsies of 24 distant metastases (10 liver, 7 lung, 4 skin, 2 ovary and 1 stomach) of non IBCs distinct from the 22 "non IBC" controls.

The tumor samples were flash-frozen in liquid nitrogen and stored at -80°C until RNA extraction. Only tumor samples containing more than 70% of tumor cells were used.

### Real-time RT-PCR

The theoretical and practical aspects of real-time quantitative RT-PCR using the ABI Prism 7700 Sequence Detection System (Perkin-Elmer Applied Biosystems) have been described in detail elsewhere [13].

The precise amount of total RNA added to each reaction mix (based on optical density) and its quality (i.e. lack of extensive degradation) are both difficult to assess. We therefore also quantified transcripts of two endogenous RNA control genes involved in two cellular metabolic pathways, namely *TBP *(Genbank accession NM_003194), which encodes the TATA box-binding protein (a component of the DNA-binding protein complex TFIID), and *RPLP0 *(NM_001002), which encodes human acidic ribosomal phosphoprotein P0. The results for each sample were normalized on the basis of the corresponding *TBP *(or *RPLPO*) mRNA content. We selected *TBP *as an endogenous control because its transcripts are moderately abundant, and because there are no known *TBP *retropseudogenes. [Retropseudogenes lead to co-amplification of contaminating genomic DNA and thus interfere with RT-PCR, despite the use of primers in separate exons.] We also selected *RPLP0 *because its transcripts are more abundant than those of *TBP*, and because this gene (better known as 36B4) is widely used as an endogenous control for northern blot analysis. Results, expressed as N-fold differences in target gene expression relative to the *TBP *(or *RPLPO*) gene, and termed "N*target*", were determined as N*target *= 2^ΔCt sample ^where the ΔCt (cycle threshold) value of the sample was determined by subtracting the average Ct value of the target gene from the average Ct value of the *TBP (or RPLP0) *gene.

The N*target *values of the samples were subsequently normalized such that the median of the non IBC N*target *values was 1.

Primers for *TBP*, *RPLP0 *and the 60 target genes (see Table 1) were chosen with the assistance of the Oligo 5.0 computer program (National Biosciences, Plymouth, MN).

**Table 1:** List of the 60 selected genes

**Gene symbols**	**Alternate symbols**	**Gene name**	**Chromosome location**	**Genbank accession number**
***NFKB genes (n = 11)***				
***NFKB1***		Nuclear factor of kappa light polypeptide gene enhancer in B-cells 1 (p105)	4q24	NM_003998
***NFKB2***		Nuclear factor of kappa light polypeptide gene enhancer in B-cells 2 (p49/p100)	10q24	NM_002502
***REL***		v-rel reticuloendotheliosis viral oncogene homolog	2p13-p12	NM_002908
***RELA***	NFKB3	v-rel reticuloendotheliosis viral oncogene homolog A (p65)	11q13	NM_021975
***RELB***		v-rel reticuloendotheliosis viral oncogene homolog B	19q13.32	NM_006509
***CHUK***	IKBKA	Conserved helix-loop-helix ubiquitous kinase	10q24-q25	NM_001278
***IKBKB***		Inhibitor of kappa light polypeptide gene enhancer in B-cells, kinase beta	8p11.2	NM_001556
***IKBKG***		Inhibitor of kappa light polypeptide gene enhancer in B-cells, kinase gamma	Xq28	NM_003639
***NLK***		Nemo like kinase	17q11.2	NM_016231
***NFKBIA***		Nuclear factor of kappa light polypeptide gene enhancer in B-cells inhibitor, alpha	14q13	NM_020529
***NFKBIB***		Nuclear factor of kappa light polypeptide gene enhancer in B-cells inhibitor, beta	19q13.1	NM_1001716
***Apoptosis (n = 12)***				
***BCL2A1***	BFL1/A1	Baculoviral IAP repeat-containing 2	15q24.3	NM_004049
***GADD45B***		Growth arrest and DNA-damage-inducible, beta	19p13.3	NM_015675
***TNFRSF10B***	TRAILR2, DR5	Tumor necrosis factor receptor superfamily, member 10b	8p22-p21	NM_003842
***FASLG***	FASL, TNFSF6	Fas ligand (TNF superfamily, member 6)	1q23	NM_000639
***BIRC4***	XIAP	Baculoviral IAP repeat-containing 4	Xq25	NM_001167
***TNFAIP3***	A20	Tumor necrosis factor, alpha-induced protein 3	6q23	NM_006290
***TRAF2***		TNF receptor-associated factor 2	9q34	NM_021138
***IER3S***		Immediate early response 3, large transcript	6p21.3	NM_003897
***IER3L***		Immediate early response 3, short transcript	6p21.3	NM_052815
***BIRC2***	c-IAP1	Baculoviral IAP repeat-containing 2	11q22	NM_001166
***MCL1S***		Myeloid cell leukemia sequence 1 (BCL2-related), short transcript	1q21	NM_182763
***MCL1L***		Myeloid cell leukemia sequence 1 (BCL2-related), large transcript	1q21	NM_021960
***Immune response (n = 15)***				
***IL1A***		Interleukin 1, alpha	2q14	NM_000575
***IL1B***		Interleukin 1, beta	2q14	NM_000576
***IL6***		Interleukin 6 (interferon, beta 2)	7p21	NM_000600
***IL12B***		Interleukin 12B (natural killer cell stimulatory factor 2, cytotoxic lymphocyte maturation factor 2, p40)	5q31.1-q33.1	NM_002187
***IL15***		Interleukin 15	4q31	NM_000585
***CCL2***	MCP-1	Chemokine (C-C motif) ligand 2	17q11.2-q21.1	NM_002982
***CCR5***		Chemokine (C-C motif) receptor 5	3p21	NM_000579
***TNFRSF11A***	RANK	Tumor necrosis factor receptor superfamily, member 11a, activator of NFKB	18q22.1	NM_003839
***TNFSF11***	RANKL	Tumor necrosis factor (ligand) superfamily, member 11	13q14	NM_003701
***TNF***		Tumor necrosis factor (TNF superfamily, member 2)	6p21.3	NM_000594
***IRF7***		Interferon regulatory factor 7	11p15.5	NM_001572
***GBP1***		Guanylate binding protein 1, interferon-inducible, 67kDa	1p22.2	NM_002053
***CD40***		CD40 antigen (TNF receptor superfamily member 5)	20q12-q13.1	NM_001250
***CD40LG***		CD40 ligand (TNF superfamily, member 5, hyper-IgM syndrome)	Xq26	NM_000074
***CD48***		CD48 molecule	1q21.3-q22	NM_001778
***Cell Proliferation (n = 8)***				
***CSF1***		Colony stimulating factor 1 (macrophage)	1p21-p13	NM_000757
***CSF1R***		Colony stimulating factor 1 receptor, formerly McDonough feline sarcoma viral (v-fms) oncogene homolog	5q33-q35	NM_005211
***CSF2***		Colony stimulating factor 2 (granulocyte-macrophage)	5q31.1	NM_000758
***CCND1***		Cyclin D1 (PRAD1: parathyroid adenomatosis 1)	11q13	NM_053056
***CCND2***		Cyclin D2	12p13	NM_001759
***CCND3***		Cyclin D3	6p21.3	NM_001760
***CCNG1***		Cyclin G1	5q32-q34	NM_004060
***SOD2***	Mn-SOD	Superoxide dismutase 2, mitochondrial	6q25.3	NM_000636
***Tumor progression (n = 10)***				
***MMP9***		Matrix metalloproteinase 9 (gelatinase B, 92 kDa type IV collagenase)	20q11.2-q13.1	NM_004994
***MMP11***		Matrix metalloproteinase 11 (stromelysin 3)	22q11.23	NM_005931
***PLAU***	UPA	Plasminogen activator, urokinase	10q24	NM_002658
***CTSB***		Cathepsin B	8p22	NM_001908
***CXCR4***		Chemokine (C-X-C motif) receptor 4	2q21	NM_003467
***CXCL12***	SDF1	Chemokine (C-X-C motif) ligand 12 (stromal cell-derived factor 1)	10q11.1	NM_000609
***ICAM1***		Intercellular adhesion molecule 1 (CD54), human rhinovirus receptor	19p13.3-p13.2	NM_000201
***VCAM1***		Vascular cell adhesion molecule 1	1p32-p31	NM_001078
***SELE***	ELAM1	Selectin E (endothelial adhesion molecule 1)	1q22-q25	NM_000450
***TNC***	HXB	Tenascin C (hexabrachion)	9q33	NM_002160
***Angiogenesis (n = 4)***				
***IL8***		Interleukin 8	4q13-q21	NM_000584
***CXCL1***	GRO1	Chemokine (C-X-C motif) ligand 1 (melanoma growth stimulating activity, alpha)	4q21	NM_001511
***VEGF***	VEGFA	Vascular endothelial growth factor	6p12	NM_003376
***PTGS2***	COX2	Prostaglandin-endoperoxide synthetase 2	1q25.2-q25.3	NM_000963

We searched the dbEST and nr databases to confirm the total gene specificity of the nucleotide sequences chosen as primers, and the absence of single nucleotide polymorphisms. In particular, the primer pairs were selected to be unique relative to the sequences of closely related family member genes or of the corresponding retropseudogenes. To avoid amplification of contaminating genomic DNA, one of the two primers was placed at the junction between two exons, if possible. In general, amplicons were between 60 and 120 nucleotides long. Gel electrophoresis was used to verify the specificity of PCR amplicons.

For each primer pair we performed no-template control (NTC) and no-reverse-transcriptase control (RT-negative) assays, which produced negligible signals (usually > 40 in Ct values), suggesting that primer-dimer formation and genomic DNA contamination effects were negligible.

The RNA extraction, cDNA synthesis and PCR conditions have been described in detail elsewhere [13].

### Statistical analysis

As the mRNA levels did not fit a Gaussian distribution, (a) the mRNA levels in each subgroup of samples were characterized by their median and range rather than their mean and coefficient of variation, and (b) relationships between the molecular markers and clinical and histological parameters were tested with the non parametric Mann-Whitney U test [23].

Hierarchical clustering was done with GeneANOVA software [24].

## Results

### mRNA expression of the 60 NF-κB-associated genes, *ESR1/ERα *and *MKI67 *in 35 IBCs and 22 non IBCs

The expression level of the 60 genes was determined individually in 35 IBCs and 22 non IBCs. Very low levels of target gene mRNA, that were detectable but not reliably quantifiable by real-time quantitative RT-PCR (Ct > 32), were observed for 4 (7%) of the 60 genes (*IL1A*, *IL6*, *IL12B*, and *CSF2*).

Thirty-five of the remaining 56 genes were significantly upregulated in the 35 IBCs relative to the 22 non IBCs (p < 0.05; Table 2). Only one gene, *BIRC4/XIAP*, was significantly down-regulated in the IBCs.

**Table 2:** List of the significantly dysregulated NF-KB-related genes in IBCs relative to non IBCs

**GENES**	**non IBC (n = 22)**	**IBC (n = 35)**	**p^b^**	**Metastases (n = 24)**	**p^c^**
**Upregulated genes in IBC**					
*CXCL12*	1,0 (0,3–8,1)^a^	6,2 (0,3–1)	**0,0000048**	1,5 (0,1–20,1)	0,2 (NS)
*PTGS2/COX2*	1,0 (0,2–28,7)	8,1 (0,2–62,6)	**0,000013**	8,9 (0,1–397,2)	**0,000018**
*CCND2*	1,0 (0,2–4,8)	2,8 (0,5–15,3)	**0,000025**	0,7 (0,1–15,3)	0,14 (NS)
*MCL1L*	1,0 (0,5–7,8)	2 (0,6–5,8)	**0,000035**	1,1 (0,2–4,7)	0,74 (NS)
*TNFAIP3/A20*	1,0 (0,4–2,7)	2,6 (0,3–12,8)	**0,000042**	0,9 (0,2–9,1)	0,83 (NS)
*GADD45B*	1,0 (0,1–12,4)	2,9 (0,1–7,3)	**0,000059**	1,1 (0,2–7,5)	0,19 (NS)
*FASLG*	1,0 (0,1–2,5)	1,8 (0,3–5,6)	**0,00011**	0,7 (0,1–7,2)	0,28 (NS)
*CXCL1/GRO1*	1,0 (0,1–108,7)	5,8 (0,2–73,4)	**0,00016**	5,3 (0,1–149,2)	**0,0026**
*MCL1S*	1,0 (0,3–3,1)	2,5 (0,5–9,2)	**0,00018**	1,4 (0,3–7,0)	0,12 (NS)
*NFKB1*	1,0 (0,4–11,1)	2,1 (0,2–8,0)	**0,00025**	0,8 (0,2–2,9)	0,92 (NS)
*CCND3*	1,0 (0,5–3,5)	1,5 (0,7–24,4)	**0,00038**	0,8 (0,3–3,3)	0,22 (NS)
*SELE*	1,0 (0,1–3,5)	3,4 (0,2–212,0)	**0,00053**	0,7 (0,0–21,7)	0,15 (NS)
*TNC*	1,0 (0,1–30,3)	2,9 (0,3–33,4)	**0,00051**	0,8 (0,1–16,2)	0,29 (NS)
*VCAM1*	1,0 (0,4–4,0)	2,1 (0,9–16,3)	**0,00051**	1,1 (0,1–7,0)	0,92 (NS)
*CD40*	1,0 (0,3–4,0)	2,7 (0,2–48,0)	**0,00076**	0,7 (0,1–9,6)	0,07 (NS)
*CSF1R*	1,0 (0,2–3,8)	2,1 (0,3–25,4)	**0,0012**	0,6 (0,1–3,1)	**0,028**
*CD48*	1,0 (0,06–8,39)	3,07 (0,05–46,98)	**0,0017**	0,54 (0,02–6,41)	**0,036**
*TNFSF11/RANKL*	1,0 (0,10–6,0)	4,2 (0,3–358,3)	**0,0019**	0,9 (0,1–19,1)	0,86 (NS)
*IER3L*	1,0 (0,1–4,8)	2,7 (0,2–19,2)	**0,0022**	1,1 (0,1–27,6)	0,80 (NS)
*TNFRSF11A/RANK*	1,0 (0,2–3,4)	1,7 (0,1–30,1)	**0,003**	1,2 (0,1–14,2)	0,66 (NS)
*RELA*	1,0 (0,3–4,5)	1,3 (0,6–4,5)	**0,0031**	0,9 (0,3–2,3)	0,65 (NS)
*CCL2/MCP-1*	1,0 (0,2–3,6)	2,1 (0,5–8,0)	**0,0039**	1,4 (0,1–10,5)	0,21 (NS)
*IKBKG*	1,0 (0,4–10,2)	1,4 (0,6–5,2)	**0,0057**	0,7 (0,2–3,7)	**0,039**
*TNFRSF10B/TRAILR2*	1,0 (0,2–2,8)	1,4 (0,3–5,2)	**0,0067**	1,1 (0,1–12,3)	0,76 (NS)
*NFKBIB*	1,0 (0,5–6,7)	1,6 (0,4–7,0)	**0,0074**	0,8 (0,3–5,3)	0,58 (NS)
*ICAM1*	1,0 (0,2–4,3)	1,9 (0,2–19,0)	**0,0083**	0,9 (0,1–28,1)	0,38 (NS)
*CD40LG*	1,0 (0,1–5,0)	2,6 (0,1–30,3)	**0,0096**	0,7 (0,1–3,1)	**0,03**
*CSF1*	1,0 (0,1–3,9)	1,8 (0,3–11,7)	**0,0096**	0,6 (0,1–2,9)	**0,044**
*PLAU/UPA*	1,0 (0,2–16,2)	2,0 (0,4–17,6)	**0,011**	0,7 (0,1–18,7)	0,21 (NS)
*NFKB2*	1,0 (0,3–3,2)	1,4 (0,4–10,8)	**0,018**	0,6 (0,2–4,7)	0,07(NS)
*IL15*	1,0 (0,00–7,08)	1,72 (0,12–9,67)	**0,021**	1,40 (0,06–8,48)	0,20 (NS)
*GBP1*	1,0 (0,17–3,71)	1,35 (0,34–7,06)	**0,032**	0,78 (0,13–9,64)	0,99 (NS)
*REL*	1,0 (0,3–3,3)	1,4 (0,2–6,6)	**0,039**	0,5 (0,2–1,5)	**0,002**
*SOD2*	1,0 (0,43–3,89)	1,34 (0,26–7,96)	**0,042**	1,16 (0,40–4,01)	0,38 (NS)
*CHUK*	1,0 (0,4–2,4)	1,1 (0,5–4,6)	**0,048**	0,7 (0,2–2,1)	0,25 (NS)

**Downregulated gene in IBC**					
*BIRC4/XIAP*	1,0 (0,2–4,0)	0,6 (0,1–1,7)	**0,026**	0,9 (0,2–15,0)	0,56 (NS)

*MKI67*	1,0 (0,4–2,8)	1,1 (0,2–9,4)	0,37 (NS)	1,1 (0,1–1327,8)	0,46 (NS)
*ESR1/ERa*	1,0 (0,0–23,2)	0,2 (0,0–7,9)	0,070 (NS)	0,1 (0,0–163,5)	0,13 (NS)

^a ^Median (range) of gene mRNA levels

^b ^Mann-Whitney U test: IBC *vs *non IBC

^C ^Mann-Whitney U test: Metastases *vs *non IBC

The 35 upregulated genes included NF-κB genes (*NFKB1*, *RELA*, *IKBKG*, *NFKBIB*, *NFKB2*, *REL*, *CHUK*) and NF-κB-regulated genes involved in apoptosis (*MCL1L*, *TNFAIP3/A20*, *GADD45B*, *FASLG*, *MCL1S*, *IER3L*, *TNFRSF10B/TRAILR2*), immune response (*CD40*, *CD48*, *TNFSF11/RANKL*, *TNFRSF11A/RANK*, *CCL2/MCP-1*, *CD40LG*, *IL15*, *GBP1*), proliferation (*CCND2*, *CCND3*, *CSF1R*, *CSF1*, *SOD2*), tumor progression (*CXCL12*, *SELE*, *TNC*, *VCAM1*, *ICAM1*, *PLAU/UPA*) or angiogenesis (*PTGS2/COX2*, *CXCL1/GRO1*).

The expression of most of the 35 genes that were upregulated in IBCs was similar in the metastases and the 22 non IBCs (Table 2). Only two (*PTGS2/COX2 *and *CXCL1/GRO1*) of these 35 genes were also upregulated in the metastases relative to the 22 non IBCs (Table 2). It is noteworthy that these two genes correspond to the two angiogenesis genes that were significantly upregulated in the 35 IBCs. Finally, six genes (*CSF1R*, *CD48*, *IKBKG*, *CD40LG*, *CSF1*, and *REL*) were slightly down-regulated in the metastases relative to the non IBCs (Table 2).

In the same set of 35 IBCs and 22 non IBCs we also examined the expression of the *ESR1/ERα *gene and the *MKI67 *gene, the latter encoding the proliferation-related antigen Ki-67. *ESR1/ERα *and *MKI67 *expression was similar in the IBCs and non IBCs, indicating that NF-kB gene upregulation in IBCs occurs in a proliferation- and ERα-independent fashion (Table 2).

The mRNA levels reported in Table 2 (calculated as described in *Materials and Methods*) show the abundance of the target relative to the endogenous control (*TBP*), used to normalize the starting amount and quality of total RNA. Similar results were obtained with a second endogenous control, *RPLP0 *(data not shown).

### Identification of a gene expression signature discriminating IBCs from non IBCs

**Figure 1:**
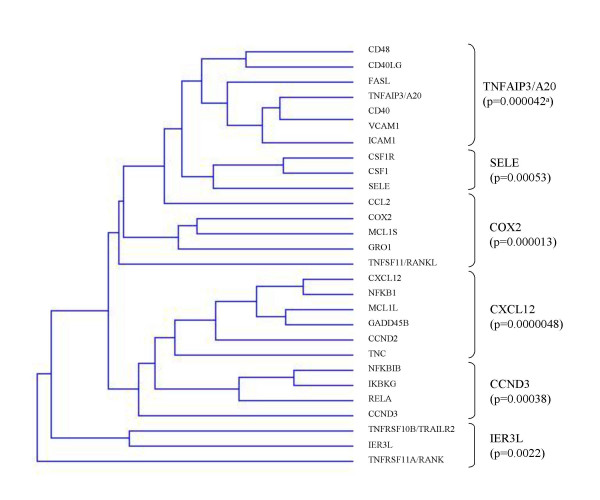
**Dendrogram of the 28 most strongly upregulated genes (p < 0.01) constructed using hierarchical clustering, according to the gene profiling of the 57 IBCs and non IBCs**. The 28 genes were categorized into 6 groups. The 6 most strongly upregulated genes (named master gene) within each group are indicated on the right (*TNFAIP3/A20*, *SELE*, *COX2*, *CXCL12*, *CCND3*, *IER3L*). ^a^: Mann-Whitney U Test (see table 2).

Hierarchical clustering analysis was used to group the 28 most strongly upregulated genes (p < 0.01) on the basis of similarity in the pattern with which their expression varied over the 57 tumors (IBCs and non IBCs). The 28 genes were thus divided into six groups (Figure 1).

**Figure 2:**
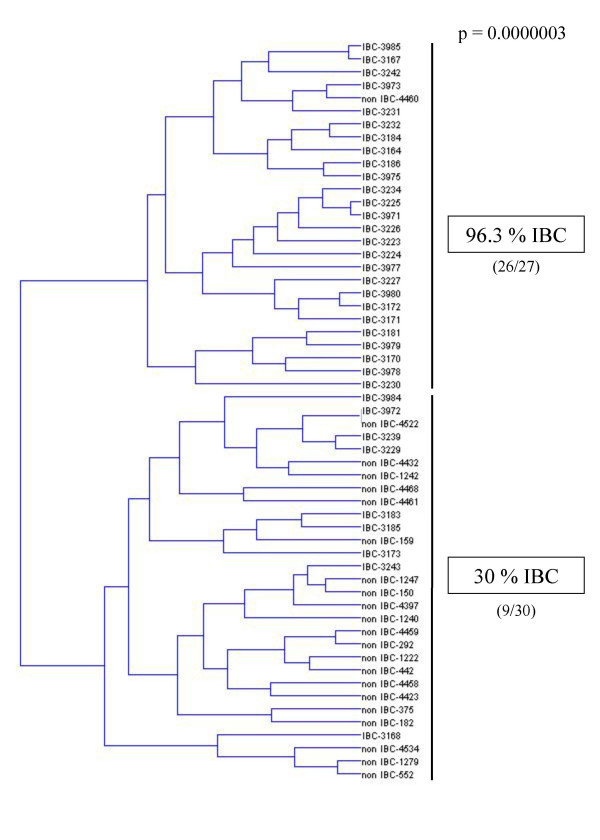
Dendrogram of the 35 IBCs and the 22 non IBC samples, constructed using hierarchical clustering, according to the expression of 6 genes, i.e. *TNFAIP3/A20*, *SELE*, *COX2*, *CXCL12*, *CCND3*, and *IER3L*. This analysis revealed two groups of tumors with 96,3% (26/27) of IBCs clustered in one group and 30% (9/30) in the second group.

We then selected six "master genes", namely *TNFAIP3/A20*, *SELE*, *COX2*, *CXCL12*, *CCND3*, and *IER3L*, corresponding to the most discriminatory genes in each group (based on the p values, cf. Table 2). Hierarchical clustering of the 35 IBC and 22 non IBC samples, based on the expression of these six master genes (see dendrogram in Figure 2) identified two groups of tumor samples, with 96.3% (26/27) of IBCs clustered in one group and 30% (9/30) in the second group (p = 0.0000003). The signature correctly classified 26 of 35 IBCs (74% sensitivity) and 21 of 22 non IBCs (95% specificity).

### mRNA expression of the 56 candidate genes according to IBC relapse status

Twenty-six (74%) of the 35 patients with IBC relapsed, a proportion in keeping with published data [25]. Comparison of the median mRNA levels of the 56 candidate genes between patients who relapsed (n = 26) and patients who did not relapse (n = 9) identified two genes -*VEGF *(p = 0.048) and *IL8 *(p = 0.042)- with lower expression in patients who relapsed.

In the same series of IBCs, we had previously identified a three-gene expression profile based on *MYCN*, *EREG*, and *SHH *(genes not involved in the NF-κB pathway) which discriminated cases with poor, intermediate and good outcome [13].

**Figure 3:**
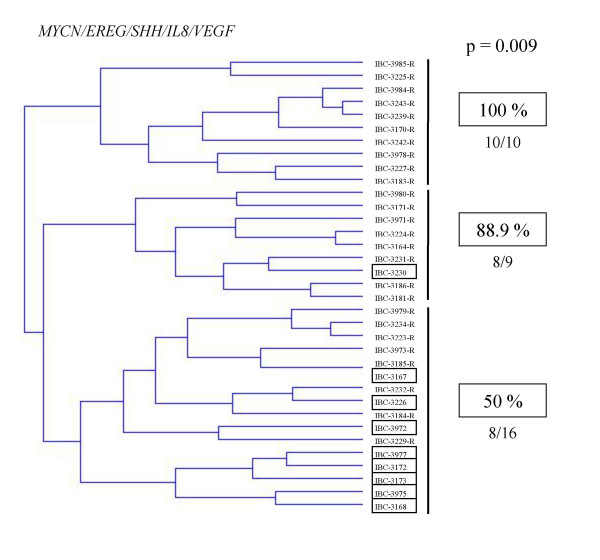
Dendrogram of 26 IBCs who relapsed (R) and 9 who did not relapse (*circled*) constructed by hierarchical clustering, according to *MYCN/EREG/SHH/IL8/VEGF *expression. The percentage of patients who relapsed are indicated on the right.

Hierarchical clustering analysis of the 35 IBCs based on a five-gene signature including the three previously identified genes (*MYCN*, *EREG*, and *SHH*) and the two genes identified here (*VEGF *and *IL8*) subdivided the patients into three groups with significantly different outcomes (p = 0.009; Figure 3): two groups of patients had very poor outcomes (respectively 100% and 88.9% relapsed), whereas 50% of the patients in the third group were free of relapse at the time of this analysis

## Discussion

IBC is a poorly understood disease with a dismal prognosis. Diagnosis is based on variously appreciated clinical signs, and prognostic factors are sorely needed. Despite multimodality treatments, the overall outcome of IBC is almost as grim as that of metastatic breast cancer [25,26]. Identification of a molecular signature might help to improve the diagnosis, as well as the prognostication and targeted therapy of IBC. The specific molecular alterations underlying IBC are largely unknown, owing to the rarity of the disease together with diagnostic uncertainties and the small size of diagnostic samples, which may have hindered past molecular studies. Moreover, previous molecular studies often grouped IBCs together with non inflammatory LABCs, whereas IBC was recently shown to be distinct from other forms of LABC, probably with different underlying molecular alterations [27,28].

Two major lines of evidence implicate NF-κB-associated pathways in IBC. First, NF-κB target genes are involved in the principal processes that are dysregulated at the clinical and molecular level in IBC, such as inflammation, proliferation and invasiveness [14,15]. Second, recent DNA microarrays studies of IBC have shown abnormal expression of some NF-κB target genes [9,10].

Real-time quantitative RT-PCR is complementary to cDNA microarray technology for tumor molecular profiling, being quantitative and also far more precise and reproducible. Moreover, RT-PCR is useful for analyzing weakly expressed genes, such as *COX2, CXCL1/GRO1, TNFSF11/RANKL *and *CD40LG *in the present study.

By using RT-PCR to compare the mRNA levels of 538 cancer genes in the same series of IBCs and non IBCs, we previously showed the upregulation of genes that mainly encoded AP1 transcription factors, but also some NF-κB target genes like *COX2 *and *VEGF *[13]. As the list of NF-κB-associated genes of interest was very incomplete in this previous study, we thoroughly scrutinized the literature on NF-κB for the present study [14,15,21]. A set of 60 major NFKB-related genes was selected for this analysis (Table 1).

The very high proportion (58%) of upregulated NF-κB-associated genes in our series of IBC was not entirely unexpected, given the functional roles of these genes in invasiveness, angiogenesis, inflammation, cell proliferation and survival. In their DNA microarray study, van Laere et al also observed a noteworthy proportion of overexpressed NF-κB target genes [10]. More recently, the same authors confirmed the involvement of some of these genes in IBC [12]. In particular, they validated by quantitative real-time RT-PCR the overexpression of 7 NF-κB-target genes (*VCAM1*, *CCR5*, *SOD2*, *CTSB*, *IRF7*, *GBP1*, and *CD48*) previously detected by them using cDNA microarrays [10,12]. We tested these seven genes in our series; *VCAM1, CD48, GBP1 *and *SOD2 *also showed a moderately significant overexpression in IBC relative to the non IBCs, whereas *CCR5, IRF7 *and *CTSB *did not (Table 2).

One very interesting finding here is that the gene expression profile of 24 distant breast metastases was quite different from that of the 35 IBCs, as all the NF-κB-associated genes were expressed at similar levels in the 24 metastatic samples and the 22 non IBCs (except for the two angiogenesis-related genes *PTGS2/COX2 *and *CXCL1/GRO1*). This further supports a strong specific link between NF-κB gene activation and the IBC phenotype.

*CXCL12*, *COX2*, *CCND2*, *MCL1L*, *TNFAIP3/A20*, and *GADD45B *were the most strongly deregulated genes in our series of IBC. *CXCL12 *and its receptor *CXCR4 *play major roles in embryogenesis, homeostasis and inflammation. They are also key regulators of carcinogenesis, acting through a wide range of mechanisms such as increased survival and proliferation of cancer cells, angiogenesis and chemoinvasion [29]. Many studies have now validated the concept that this receptor-ligand pair strongly influences metastasis, in particular by directing the migration of cancer cells to sites of metastasis. The role of *COX2 *in mammary oncogenesis is also well established, and clinical trials of COX2 inhibitors like celecoxib are ongoing in breast cancer [30]. However, *COX2 *was also upregulated in the breast-cancer metastases and was thus not specifically dysregulated in IBC, contrary to most of the other NF-κB-associated genes tested here (Table 2). We observed an overexpression of three anti-apoptotic genes: i.e. *MCL1L*, *TNFAIP3/A20*, and *GADD45B*. Van Laere et al. also observed elevated *GADD45B *expression in IBC samples [10]. The activation of NF-κB-dependent anti-apoptotic genes may promote IBC tumorigenesis, as it has been shown in other inflammation-associated tumor types [31]. However, what matters in IBC is probably not the overexpression of a particular NF-κB-associated gene but rather the activation of the entire NF-κB pathway.

We think that one of the best ways to identify specific molecular alterations in IBC is to use "stage-matched" non inflammatory breast tumors as controls, and to strictly select patients with IBC. This approach can point out genes that are specifically associated with the IBC phenotype rather than with a poor prognosis in general. Applying these criteria, we identified a six-gene signature (*TNFAIP3/A20*, *SELE*, *COX2*, *CXCL12*, *CCND3*, *IER3L*) discriminating IBC from non IBC. However, nine IBCs were misclassified as non IBCs, even though they did not differ from the other 26 IBCs in terms of patient age, histological grade, hormone receptor status, PEV classification or prognosis (data not shown). The 6-gene signature was tested on an independent series of 37 IBCs and 44 non IBCs studied using cDNA microarrays [9]. Two genes (*CCND3 *and *SELE*) significantly discriminated the 37 IBC from the 44 non IBCs (p = 0,01; Bertucci F, personal data). The other four genes (*TNFAIP3/A20*, *COX2*, *CXCL12*, and *IER3L*) were not expressed at significant levels (> 2 × background signal in at least 50% of all tumor samples). Unfortunately, we could not test the signature at the protein level because no more paired paraffin-embedded tumor samples were available for immunohistochemistry (IHC) analysis. It will be important to perform the IHC on an independent prospective series of IBC samples.

Contrary to some DNA anomalies that we have previously observed in IBC by means of allelic imbalance analysis, we found no significant difference here in NF-κB-associated gene expression levels between PEV2 tumors (localized inflammation) and PEV3 tumors (extensive inflammation and poorer prognosis than PEV2 tumors) [32]. In particular, our previous study showed that 17q21 deletion was more frequent in PEV3 tumors. However, none of the genes found to be upregulated in the present study is located in this region. Finally, it should be borne in mind that several genes may be altered in all IBCs while others are specifically altered in certain IBC subtypes.

We also examined the prognostic significance of NF-κB-associated genes in IBC. Although the statistical significance was weak, we found that lower *VEGF *and *IL8 *expression was associated with relapse. This is surprising, as both genes promote angiogenesis. Furthermore, a five-gene expression profile with *VEGF*, *IL8 *and the three genes (*MYCN*, *SHH*, and *EREG*) that we previously showed to be associated with outcome in the same series of IBCs [13] clearly delineated two subgroups of IBC with high (near 100%) and low (50%) relapse rates (Figure 3).

## Conclusion

These results demonstrate that the NF-κB pathway plays a major role in IBC. Activation of NF-κB-associated genes appears to contribute to the IBC phenotype and may prove to be of prognostic significance. Furthermore, upregulated NF-κB-related genes might serve as novel therapeutic targets in IBC. It is noteworthy that several NF-κB inhibitors are known to have antitumoral activity in breast cancer [33,34] and that one has been shown to halt the growth of IBC xenografts, either alone or in combination with an anthracycline [35,36].

## Competing interests

The author(s) declare that they have no competing interests.

## Authors' contributions

FL helped design the study and analyze data, and wrote the manuscript. SV and CA did RNA extraction, cDNA synthesis and QRT-PCR. ME and MM participated in patients' selection and treatment. RL collected specimens and helped design the study. IB designed the study and analyzed data. All authors read and approved the final manuscript.

## Pre-publication history

The pre-publication history for this paper can be accessed here:


